# Müllerian Duct Anomalies and Anti-Müllerian Hormone Levels in Women With Polycystic Ovary Syndrome

**DOI:** 10.7759/cureus.43848

**Published:** 2023-08-21

**Authors:** Min Yang, Fang Zhang, Kaiqi Wu, Dong Yu, Yi Zhang, Yun Liao, Gufeng Xu, Yue Wang

**Affiliations:** 1 Department of Ambulatory Surgery, Women’s Hospital, Zhejiang University School of Medicine, Hangzhou, CHN; 2 Department of Reproductive Endocrinology, Women’s Hospital, Zhejiang University School of Medicine, Hangzhou, CHN; 3 Department of Clinical Laboratory, Women’s Hospital, Zhejiang University School of Medicine, Hangzhou, CHN; 4 Department of Sonography, Women’s Hospital, Zhejiang University School of Medicine, Hangzhou, CHN

**Keywords:** uterine malformation, unicornuate uterus, polycystic ovary syndrome, müllerian duct anomalies, anti-mullerian hormone

## Abstract

Background: Significant associations between the presence of polycystic ovary syndrome (PCOS) and uterine anomalies have been reported. It is unclear whether high anti-Müllerian hormone (AMH) levels coexist with the development of uterine malformations in women with PCOS. This study sought to investigate the association between Müllerian duct anomalies and anti-Müllerian hormone (AMH) levels in women with PCOS.

Methods: In this retrospective cohort study, the records of 1,391 women with PCOS were analyzed. The cohort was divided into a low-AMH group (n = 700) and a high-AMH group (n = 691), based on an AMH cutoff value of 8.45 ng/ml. Müllerian duct anomalies were classified into four subtypes based on three-dimensional ultrasonography: septate uterus, bicornuate uterus, uterus didelphys, unicornuate uterus, and arcuate uterus. The primary outcome was the overall incidence of Müllerian duct anomalies. The secondary outcome was the prevalence of the abovementioned specific types of Müllerian duct anomalies. The prevalence of Müllerian duct anomalies was analyzed using the chi-squared test or Fisher’s exact test.

Results: Among the patients with PCOS, the prevalence of unicornuate uterus anomalies was higher in the high-AMH group than in the low-AMH group (1.0% vs. 0.1%, P = 0.04). No statistically significant difference in the overall incidence of uterine malformations was found between the two AMH groups (4.3% vs. 5.7%, P = 0.22).

Conclusions: Our study confirmed a higher prevalence of unicornuate uterus in PCOS women with high AMH levels. Clinicians might decide to investigate the possibility of a unicornuate uterus in PCOS women with high AMH levels.

## Introduction

Polycystic ovary syndrome (PCOS) is common in women, and its pathophysiology appears to be polygenic and multifactorial. It is characterized by abnormal metabolic, endocrine, and reproductive factors, and is clinically diagnosed depending on anovulation/oligo-ovulation, hyperandrogenism, and polycystic ovaries. Anti-Müllerian hormone (AMH), also called Müllerian inhibiting factor, is secreted by granulosa cells of the small antral and pre-antral ovarian follicles [1]. AMH restrains the recruitment of primordial follicles and contributes to ovulatory disturbances in women [2]. Several studies have confirmed that the serum concentration of AMH is a strong indicator of PCOS [2,3]. AMH plays important roles in sexual gonadal differentiation, development, and functions in women with PCOS [4,5]. In animal models, exposure to high AMH levels in utero reprograms the fetus and induces PCOS in adulthood [6].

Müllerian duct anomalies are a group of disorders that occur when the Müllerian ducts fail to develop normally. The Müllerian ducts are the embryological precursors of the female reproductive tract, including the fallopian tubes, uterus, cervix, and the upper part of the vagina. Müllerian duct anomalies consist of a group of miscellaneous deviations from normal duct anatomy. The most common Müllerian duct anomalies include a unicornuate, didelphys, bicornuate, septate, or arcuate uterus. Müllerian duct anomalies are the result of failure of abnormal formation, fusion, or resorption of the Müllerian ducts during fetal life, and can result in repeated pregnancy loss, preterm delivery, persistent menstrual irregularity, and dysmenorrhea [7,8]. Some studies have reported significant associations between the presence of PCOS and Müllerian duct anomalies [9-11]. However, whether AMH levels play a role in the development of Müllerian duct anomalies in PCOS women is unclear.

To date, no study has investigated whether AMH is associated with Müllerian duct anomalies in women with PCOS. We hypothesized that in women with PCOS, the serum concentration of AMH would be associated with the prevalence of Müllerian duct anomalies. In this retrospective cohort study, we investigated this hypothesis.

## Materials and methods

Institutional review board approval

This study was reviewed and approved by the Ethics Committee of the Women’s Hospital School of Medicine, Zhejiang University (IRB-20210110-R). This was a retrospective study with all data extracted from medical records during routine clinical practice; therefore, the need to obtain informed patient consent was waived.

Selection criteria

Data of patients who underwent inpatient surgery at the Women’s Hospital School of Medicine, Zhejiang University, between March 2017 and December 2020 were collected. All clinical records and information were anonymized prior to the analysis. Patients were included if they met all of the following criteria: (i) age between 18-40 years, (ii) PCOS diagnosis based on the European Society of Human Reproduction and Embryology Recommendations for PCOS 2018 [12], (iii) three-dimensional ultrasonography of the uterus performed at least once. The exclusion criteria were as follows: correction of any Müllerian duct anomalies before the execution of the three-dimensional ultrasonography of the uterus.

**Figure 1:**
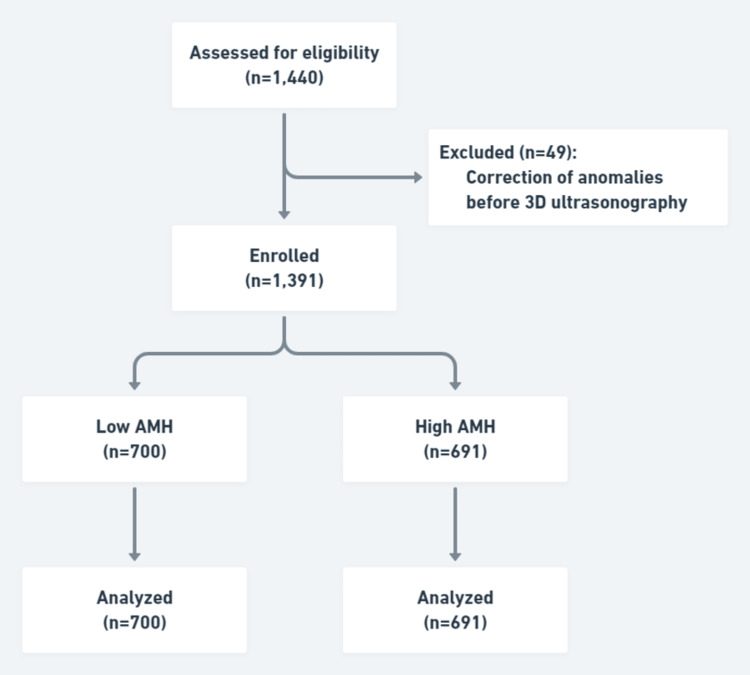
Workflow of the study. AMH, anti-Müllerian hormone.

All data were extracted from the inpatient database of the Women’s Hospital School of Medicine, Zhejiang University. The workflow of the study is provided in Figure 1.

Determination of Müllerian duct anomalies

Müllerian duct anomalies are those structural anomalies caused by errors in the development of the Müllerian duct, which is the embryonic structure that develops into the female reproductive tract. Müllerian duct anomalies usually lead to structural anomalies of the uterus. Three-dimensional ultrasonography was performed by medical sonographers with over 8 years of experience in order to determine Müllerian duct anomalies. Based on the criteria defined by the American Fertility Society system, Müllerian duct anomalies in this study were classified into five subtypes: septate uterus, bicornuate uterus, uterus didelphys, unicornuate uterus, and arcuate uterus [13].

Exposure and outcomes

Patients were assigned to two study groups based on the median AMH level in our study population: the high-AMH group (serum concentration of AMH＞8.45 ng/ml), and the low-AMH group (serum concentration of AMH ≤ 8.45 ng/ml). The primary outcome was the overall incidence of Müllerian duct anomalies. The secondary outcome was the prevalence of specific types of müllerian duct anomalies, namely septate uterus, bicornuate uterus, uterus didelphys, unicornuate uterus, and arcuate uterus.

Statistical analysis

Data were analyzed using SPSS Statistics version 22.0 (IBM, Armonk, NY, USA). The prevalence of uterine abnormalities was compared between the low- and high-AMH groups. Continuous variables, including age, body mass index (BMI), gravidity, parity, AMH, and testosterone were analyzed using Student’s t-test, whereas categorical variables, including the prevalence of Müllerian duct anomalies, were analyzed using the chi-squared test or Fisher’s exact test.

## Results

A total of 1,391 women diagnosed with PCOS were enrolled, including 700 in the low-AMH group and 691 in the high-AMH group. Compared to patients in the low-AMH group, those in the high-AMH group had a lower BMI (22.3 ± 2.9 kg/m2 vs. 23.1 ± 3.9 kg/m2, P < 0.05), and higher serum testosterone (1.4 ± 0.7 nmol/L vs. 1.2 ± 0.7 nmol/L, P < 0.05) levels. No significant differences between the two groups were identified for other clinical features (Table 1).

**Table 1:** Baseline characteristics. Data were mean (standard deviation). BMI, body mass index; AMH, anti-Müllerian hormone. Variables were calculated by Student t-test.

Variables	Low AMH (n=700)	High AMH (n=691)	P-value
Age, year	28.1±3.7	28.0±3.7	0.59
BMI, kg/m^2^	23.1±3.9	22.3±2.9	<0.01
Gravidity	0.7±1.1	0.6±0.9	0.05
Parity	0.1±0.4	0.1±0.4	0.96
AMH, ng/ml	5.9±1.6	12.7±3.7	<0.01
Testosterone, nmol/l	1.2±0.7	1.4±0.7	<0.01
Menstrual cycle length, n (%)			<0.01
<26 days	30 (4.3%)	41 (5.9%)	
26-34 days	51 (7.3%)	37 (5.3%)	
35-41 days	141 (20.1%)	89 (12.8%)	
6 weeks to 3 months	279 (39.8%)	271 (49.3%)	
>3 months	200 (28.5%)	260 (37.2%)	

Compared to women in the low-AMH group, those in the high-AMH group had a higher prevalence of unicornuate uterus anomalies (1.0% vs. 0.1%, P = 0.04). No other anomalies were significantly different between the two groups. There was no statistical difference in the overall incidence of uterine malformations between the two groups (4.3% vs. 5.7%, P = 0.22) (Table 2).

**Table 2:** Prevalence of uterine anomalies. Data were n, %. Variables were calculated with chi-squared tests or Fisher’s exact test. AMH, anti-Müllerian hormone.

Uterine anomalies	Low AMH (n=700)	High AMH (n=691)	P-value
Overall	30, 4.3%	40, 5.7%	0.22
Septate uterus	15, 2.1%	16, 2.3%	0.85
Bicornuate uterus	1, 0.1%	3, 0.4%	0.37
Uterus didelphys	1, 0.1%	0, 0.0%	1.00
Unicornuate uterus	1, 0.1%	7, 1.0%	0.04
Arcuate uterus	12, 1.7%	14, 2.0%	0.70

## Discussion

In this study, we evaluated the association between Müllerian duct anomalies and AMH levels in women with PCOS. Among women with PCOS, we found a significantly (10-fold) higher prevalence of unicornuate uterus anomalies in the high-AMH group compared to the low-AMH group.

Recent studies have suggested that changes in the intrauterine environment, such as excessive androgen [14,15] or AMH [6] exposure during the embryonic period, may be partially responsible for the development of PCOS [16]. One animal study has shown that prenatal AMH exposure could lead to PCOS in adulthood [6]. On the other hand, AMH signals the prenatal regression of the Müllerian ducts in the fetus. AMH and testosterone act synergistically to suppress the development of Müllerian ducts and promote the development of Wolffian ducts in male embryos, whereas in the absence of these two hormones the Müllerian ducts develop into female genitalia [17,18]. Our study confirmed a correlation between the prevalence of Müllerian duct anomalies due to abnormal organogenesis of the uterus and high AMH levels in patients with PCOS, inferring that the association between PCOS and some types of Müllerian duct anomalies, such as unicornuate uterus, could be explained by certain developmental defects that contribute to both PCOS and Müllerian duct anomalies. Considering that AMH and testosterone play an important role during the early degeneration of Müllerian ducts [19-21], as well as the strong heritability of PCOS [15], we suggest that high prenatal AMH exposure leads to both PCOS and Müllerian duct anomalies in female offspring.

Moreover, in animal models of PCOS, exposure to high AMH levels in utero reprograms the fetus and induces PCOS in adulthood [6]. In adult women with PCOS, the severity of the reproductive dysfunction correlates positively with serum AMH levels [22,23], and AMH could also serve as an indicator of PCOS severity. Taken together, a high level of AMH (indicating severe PCOS) and Müllerian duct anomalies may be two consequences of high prenatal AMH exposure.

The development of the Müllerian duct typically involves three stages: organogenesis, fusion, and septal resorption or tabularization. Disruption of development at any stage can lead to uterine abnormalities. Organogenesis abnormalities include uterine hypoplasia, such as Mayer-Rokitansky-Kuster-Hauser syndrome and unicornuate uterus. Abnormal fusion, septal absorption, or tabularization can lead to uterus didelphys, bicornuate, septate, and arcuate uterus [24]. The risk factors for Müllerian duct anomalies remain unclear but include hypoxia during pregnancy, the use of diethylstilbestrol, exposure to hazardous waste, ionizing radiation, and viral infections [25-27]. The specific mechanism by which the uterine septum regresses is reported to be associated with apoptosis, which is further regulated by BCL2 [28].

A major limitation of this study is that the number of patients with Müllerian duct anomalies was small, and adjustments for confounding factors, such as age and parity, could not be made. Moreover, the number of patients with bicornuate uterus and uterus didelphys were too few (2 and 4, respectively), and therefore the results of these two types of anomalies may have not reached statistical significance. Further research should expand the study population to address these limitations. Another limitation is that patients with amenorrhea due to uterine hypoplasia were not included in the study, because of the need to measure the menstrual cycle for PCOS diagnosis. Additionally, some data were unavailable, which is inevitable in retrospective studies. This may also have limited the significance of the results obtained in this study.

## Conclusions

In conclusion, the present study confirmed a higher prevalence of unicornuate uterus in PCOS women with high AMH levels. These findings could potentially influence the diagnostic process for women with PCOS. If a woman with PCOS presents with high AMH levels, clinicians might decide to investigate the possibility of a unicornuate uterus or other Müllerian duct anomalies. This study expands our understanding of the relationships among PCOS, AMH, and Müllerian duct anomalies, and could inform clinical practice and guide future research. It is essential to note, however, that the relationship uncovered in this study is correlational and does not prove causation. Additional research would be needed to firmly establish the reasons behind these associations.
